# A Refractory Electrical Storm after Acute Myocardial Infarction: The Role of Temporary Ventricular Overdrive Pacing as a Bridge to ICD Implantation

**DOI:** 10.3390/pathophysiology31010004

**Published:** 2024-01-14

**Authors:** Mijo Meter, Josip Andelo Borovac

**Affiliations:** 1 Cardiovascular Diseases Department, University Hospital of Split (KBC Split), Spinciceva 1, 21000 Split, Croatia; mijometer05@gmail.com; 2 Department of Pathophysiology, University of Split School of Medicine, Soltanska 2, 21000 Split, Croatia

**Keywords:** acute coronary syndrome, electrical storm, case report, antiarrhythmic therapy, ventricular overdrive pacing, VOP, ICD, myocardial infarction, bridge-to-ICD, medical management

## Abstract

An electrical storm (ES) is defined as the presence of at least three episodes of sustained ventricular tachycardia or ventricular fibrillation within 24 h. This patient had a previously known arterial hypertension, type II diabetes mellitus, and chronic kidney disease and has presented to the Emergency Department (ED) with symptoms of retrosternal chest pain lasting for several hours prior. The initial 12-lead electrocardiogram revealed ST segment elevation in the anterior leads (V1–V6). Emergent coronary angiography revealed an acute occlusion of the proximal left anterior descending artery (pLAD) and percutaneous coronary intervention was performed with successful implantation of one drug-eluting stent in the pLAD. On day 8 of hospitalization, the patient developed a refractory ES for which he received 50 DC shocks and did not respond to multiple lines of antiarrhythmic medications. Due to a failure of medical therapy, we decided to implant a temporary pacemaker and initiate ventricular overdrive pacing (VOP) that was successful in terminating ES. Following electrical stabilization, the patient underwent a successful ICD implantation. This case demonstrates that VOP can contribute to hemodynamic and electrical stabilization of a patient that suffers from refractory ES and this treatment modality might serve as a temporary bridge to ICD implantation.

## 1. Introduction

An electrical storm (ES) indicates a state of life-threatening cardiac electrical instability. It is defined as the presence of at least three distinct episodes of sustained ventricular tachycardia or VF in the last 24 h [1]. We present a case of a 63-year-old male who presented with acute anterior myocardial infarction (AMI) and ES refractory to multiple antiarrhythmic therapies including propranolol, amiodarone, and lidocaine. Eventually, after temporary ventricular overdrive pacing (VOP) was initiated, the electrical storm was successfully terminated.

## 2. Case Report

**Figure 1:**
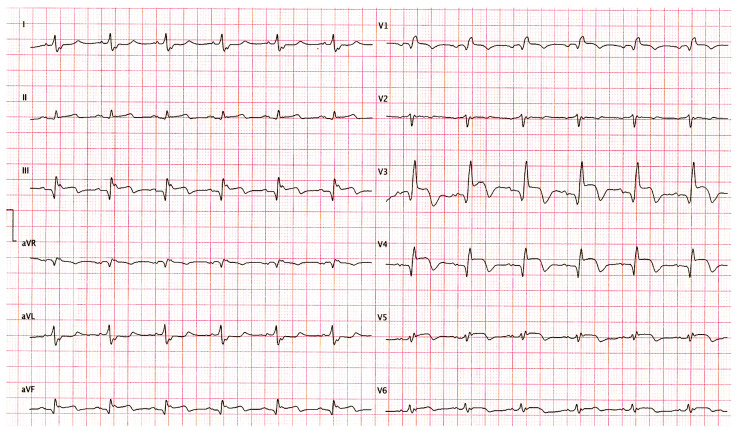
Electrocardiogram (ECG) during admission at the Emergency Department revealed elevation of the ST segment in the V1–V6 leads consistent with acute anterior myocardial infarction.

A 63-year-old male with previously known arterial hypertension, diabetes mellitus type II, and chronic kidney disease presented to the Emergency Department (ED) with symptoms of retrosternal chest pain and diaphoresis lasting for several hours before admission. The initial electrocardiogram at the ED revealed elevation of the ST segment in the V1–V4 leads, confirming the diagnosis of acute anterior myocardial infarction (Figure 1). In the physical examination, bilateral crackles in the basal portions of the lungs with mild pretibial edema were determined. Laboratory investigations revealed a normal complete blood count with increased troponin I (200 ng/L) and creatinine (130 micromoles/L) levels. Soon after admission into the Intensive Coronary Care Unit, the patient became tahydyspnoic with the development of acute respiratory insufficiency.

**Figure 2:**
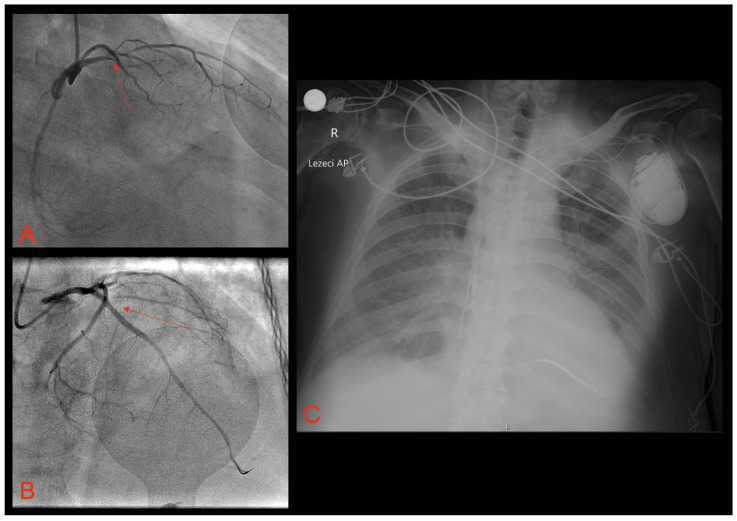
(**A**) Coronary angiography showing acute occlusion of the proximal left anterior descending artery (LAD); (**B**) coronary angiography showing established TIMI 3 flow through previously occluded LAD, following percutaneous coronary intervention and drug-eluting stent implantation; (**C**) antero-posterior chest X-ray showing the implanted ICD. Red arrows show a culprit lesion in the LAD.

We decided to perform endotracheal intubation and invasive mechanical ventilation was started. Emergent coronary angiography showed acute occlusion of the proximal left anterior descending artery (Figure 2A). After multiple balloon dilatations with semi-compliant balloons (2.0 × 20 mm and 3.0 × 30 mm), a one 3.5 × 38 mm drug-eluting stent (DES) was successfully deployed with the optimal angiographic result and TIMI 3 flow (Figure 2B).

After the procedure, the patient was hypotensive and intravenous dobutamine was started at an infusion rate of 5 mcg/kg/min with intravenous noradrenaline at an infusion rate of 6.6 mcg/min. Echocardiography showed a severely reduced left ventricular ejection fraction (LVEF) with an estimated LVEF of 30% with the Simpson Biplane method with signs of initial apical aneurysmal formation. Transthoracic echocardiography also revealed grade II diastolic dysfunction and mild-to-moderate mitral regurgitation and slightly increased left ventricular end-diastolic pressure (LVEDP) with an estimated E/e’ ratio of 17.8. Due to refractory oliguria, we started with renal replacement therapy (RRT) through the dialysis catheter placed in the right internal jugular vein. On day 7, the patient was successfully extubated, and previously applied inotropic and vasoactive therapy was de-escalated.

**Figure 3:**
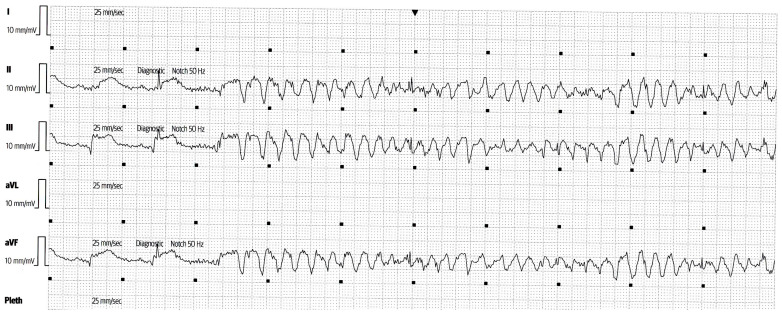
Electrocardiogram (ECG) from the telemetry system in the Coronary Care Unit (CCU) showing one of the many episodes of refractory ES (polymorphic VT/VF) despite optimal antiarrhythmic therapy.

On day 8 of hospitalization, he developed a refractory ES for which he received more than 50 DC shocks. An example of one of numerous VT runs, as captured using ICCU telemetry, is shown below in Figure 3.

A comprehensive antiarrhythmic therapy including 5 mg of intravenous metoprolol every 5 min up to three doses, an amiodarone bolus dose of 150 mg over 10 min followed by continuous infusion of 1200 mg in 24 h, a lidocaine bolus dose of 1 mg/kg, and a repeat bolus dose of 0.5 mg/kg followed by continuous infusion of 20 mcg/kg/min during 24 h was initiated (Table 1). A bolus infusion of 2 g of intravenous magnesium-sulfate was also applied. We introduced sedation with a propofol bolus dose of 50 mg (intravenous) followed by continuous infusion of 100 mcg/kg/min and decided to start again with the mechanical ventilation in order to suppress increased catecholaminergic drive.

Despite these therapeutic efforts, the patient still had episodes of polymorphic ventricular tachycardia. In order to suppress refractory ventricular arrhythmia, it was decided to perform temporary VOP. This was accomplished by using a temporary pacemaker, programmed at 90 bpm, with an active fixation lead placed in the right ventricle, using the right transfemoral approach. After the placement of the temporary pacemaker, acute hemodynamic and electrical stability was achieved.

In order to exclude possible acute stent thrombosis, we repeated invasive coronary angiography, which revealed no signs of stent thrombosis with normal flow through the previously implanted DES. The electrolytes were within a normal range and there were no metabolic disturbances observed in the laboratory workup. The measured QTc interval was within the reference range. After initial stabilization, metoprolol was replaced by propranolol peroral in a dose of 40 mg twice a day. After five days of VOP, we decided to implant the subcutaneous temporary pacing via the right subclavian vein, programmed at 85 bpm. Finally, following electrical stabilization, seven days after implantation of subcutaneous temporary pacing, the patient underwent an ICD implantation (Figure 2C) with the pacing rate programmed at 60 bpm, and two zones of tachycardia detection and corresponding therapies (VT zone at 170 bpm; VF zone at >210 bpm).

On the 25th day of hospitalization, he developed acute respiratory insufficiency, which required non-invasive mechanical ventilation (NIV). A chest X-ray showed diffuse bilateral consolidations and his nasal swab for SARS-CoV-2 infection was found to be positive. On the first day of illness, the remdesivir in a dose of 200 mg (intravenous), and on the next two days in a dose of 100 mg (intravenous), was applied. After five days, he was successfully weaned from the NIV with no signs of respiratory insufficiency. After 40 days of hospitalization, he was discharged from the hospital with optimal medical therapy including aspirin (100 mg per day), ticagrelor (90 mg twice per day), amiodarone (200 mg twice per day), metoprolol (50 mg twice per day), eplerenone (25 mg once a day), empagliflozin (10 mg once a day), and furosemide (20 mg twice a day). Repeated echocardiography at discharge showed an LVEF of 35% with the Simpson Biplane method and now only mild mitral regurgitation with a decreased E/e’ ratio and no signs of apical thrombus formation. 

## 3. Discussion

The incidence of sustained ventricular arrhythmias (VAs) in acute coronary syndromes (ACSs) is 5–10%. In the context of ischemia, VPC, VT, and VF can be secondary to an automatic or re-entrant mechanism [2,3]. Studies have shown that in only 10–25% of patients with the ES, clear precipitating causes were identified [4]. It is also estimated that ES occurs in about 10 to 20% of patients with implanted ICD [5]. Electrolyte imbalance, acute ischemia, exacerbation of heart failure, adjustment of or non-compliance to antiarrhythmic medications, and recent introduction to biventricular pacing have been identified as potential triggers [6]. The ES onset is more frequently preceded by monomorphic VT than polymorphic VT, but when polymorphic VT or VF is the cause of ES, it is usually related to the acute myocardial infarction and advanced heart failure [7]. Electrical remodeling and abnormal impulse propagation could be potential sources of ES, especially when they are accompanied by a reduced LVEF less than 25% and a QRS duration more than 120 msec [8]. The sympathetic nervous system can be a significant contributing factor in initiation and maintenance of ES, especially in conditions such as the congenital long QT syndrome (LQTS) and catecholaminergic polymorphic VT (CPVT) [9]. Furthermore, from the prognostic standpoint, patients who develop ES are more likely to die and they might have up to a two-fold higher risk of all-cause mortality, especially in the follow-up period of 3 months, while some studies showed an increased mortality rate among patients with ES even during the longer follow up extending to 3 years after the index event [5]. The reasons for increased mortality in this patient population are not fully elucidated; however, it seems that mortality is more significantly driven by the progression of cardiac dysfunction following discharge from the hospital as opposed to the potential arrhythmic instability inherently attributed to ES [5]. 

According to the most recent European Society of Cardiology guidelines for the management of ventricular arrhythmias and sudden cardiac death, overdrive pacing with a slightly higher rate than the baseline rhythm can be useful to temporarily suppress slow recurrent/incessant VTs, although this was not endorsed with the formal level of recommendation [10]. According to these guidelines, modes of treatment (from highest to least endorsed) in case of recurrent ventricular arrhythmias should be the following: (a) catheter ablation; (b) deep sedation/intubation; (c) autonomic modulation; (d) mechanical circulatory support; and (e) overdrive pacing.

To our knowledge, temporary use of VOP has been previously described by Magdi et al. in a patient that underwent PCI and had a large anterolateral AMI for the purpose of control of the resistant arrhythmia [11]. Similarly, although a different case was described by Kurisu and colleagues, in this case report, ES occurred seven days after acute anterior myocardial infarction and stent implantation and it was also complicated with ventricular fibrillation that occurred due to subacute stent occlusion, thus requiring emergent balloon angioplasty [12]. Authors report on the use of atrioventricular sequential overdrive pacing in the total duration of 25 h after which the malignant arrhythmia never recurred. On the other hand, according to the study by Nielsen et al., it should be noted that atrioventricular pacing can also reduce global mean myocardial blood flow as well as the LVEF. Thus, in patients with initially reduced LVEF, overdrive ventricular pacing may also lead to further left ventricular desynchronization, especially during fast cardiac rhythms, which can result in patients’ hemodynamic instability [13].

Temporary (atrial) overdrive pacing may help to interrupt an incessant or re-occurring VA, especially in conditions such as Brugada syndrome and early repolarization syndrome, by preventing PVCs from occurring and reducing early after-depolarization [14,15]. For example, Chalupova et al. reported on the use of temporary atrial overdrive pacing that allowed for complete suppression of ventricular ectopy in a patient with non-ST segment elevation myocardial infarction who experienced ES four days after PCI [16]. Kamakura et al. reported the case of VF after acute myocardial infarction refractory to multiple medical therapies, which was successfully terminated only by atrial pacing at 100 beats per minute. These beneficial effects could be explained due to the fact that atrial overdrive pacing at a high rate in that case could block retrograde conduction into the Purkinje system, suppressing re-entry involving the Purkinje network and VF initiation [17].

Antiarrhythmic medications are the cornerstone of ES management and their administration is required as a part of initial resuscitative measures as we summarize in Table 2 [18,19]. Only after pharmacologic means are shown to be ineffective in mitigating tachyarrhythmia, VOP should be considered. Thus, as suggested by Kurisu et al., temporary VOP might be a feasible and simple treatment option to mitigate drug-resistant ES associated with AMI and should be performed in the early stage [12].

Current guidelines also recommend deep sedation as a therapeutic option in ES refractory to antiarrhythmic drugs to reduce sympathetic overactivity involved in ES initiation and maintenance [18]. Due to the implicated role of sympathetic nervous system hyperactivity in a refractory ventricular storm, a stellate ganglion blockade might be an efficacious invasive and non-pharmacological treatment option for the management of ES as it significantly reduces ventricular arrhythmia burden and a number of external and ICD shocks [20]. Furthermore, this treatment modality has been supported by the ESC guideline class of recommendation IIb, level of evidence C [10]. 

In some cases, ES patients might experience episodes of monomorphic VT based on re-entry. Therefore, catheter ablation, targeting the substrate in which re-entry has formed, is an important treatment option for the ES in this subset of patients [21]. Catheter ablation should also be considered in patients with recurrent symptomatic episodes of PVT or VF triggered by a similar PVC. Ablation of the focal Purkinje-related triggers frequently arising from the scar border zone at the left ventricular septum appears to be associated with short- and long-term freedom from a recurrent VF storm [22]. Current guidelines recommend catheter ablation in patients presenting with incessant VTs or ES due to VT refractory to multiple antiarrhythmic therapies [10]. 

Compared to medical therapy, catheter ablation reduces the number of subsequent VT episodes especially when VT ablation is performed within one month of an ES [23]. Stereotactic arrhythmia radioablation (STAR) as a noninvasive, effective, and well-tolerated treatment may be a suitable alternative method for patients with cardiac arrhythmia who are resistant or intolerant to catheter ablation [24]. Eventually, if all these therapeutic measures failed in the acute termination of ES, mechanical circulatory support, urgent catheter ablation, or neuraxial modulation are potential options in these situations to achieve hemodynamic and electrical stabilization [25,26,27]. Other, rarely employed but feasible, therapeutic approaches in clinical practice such as thoracic epidural anesthesia with infusion of a long-acting local anesthetic such as bupivacaine or ropivacaine into the T1–T2 or T2–T3 epidural space can also serve as a bridge to other therapeutic solutions or when catheter ablation fails to terminate the arrhythmia [28]. Some surgical avenues such as cardiac sympathetic denervation, which include the surgical removal of the lower half of the left or bilateral stellate ganglia and the T2–T4 level, can reduce the burden of implantable cardioverter–defibrillator (ICD) shocks in up to 90% of patients according to a study by Vaseghi and colleagues [29].

## 4. Conclusions

A temporary VOP can serve as a feasible and effective therapeutic modality in patients presenting with ES following acute myocardial infarction refractory to multiple antiarrhythmic drugs. In these circumstances, VOP can be used as a temporary bailout therapy for acute hemodynamic and electrical stabilization and serve as a bridge to other therapeutic modalities, such as ICD implantation. 

## Figures and Tables

**Table 1:** Drugs with dose and type of administration that are indicated and were used for the management of our patient with the intention to terminate the electrical storm.

Drug	Dose	Administration
Amiodarone	150 mg bolus + 1200 mg continuous infusion during 24 h	intravenous
Lidocaine	Bolus dose of 1 mg/kg, with repeat bolus dose of 0.5 mg/kg, followed by the continuous infusion of 20 mcg/kg/min during 24 h	intravenous
Metoprolol	5 mg intravenously every 5 min; up to 3 doses	intravenous
Magnesium-sulphate	2 g bolus dose	intravenous
Propofol	50 mg bolus dose + continuous infusion of 100 mcg/kg/min	intravenous
Propranolol	40 mg twice a day after acute stabilization	oral

**Table 2:** Summary of the antiarrhythmic drugs that have an indicated use for the ES—reproduced and modified based on the work of Kowlgi et al. [19].

Drug	Dose
Amiodarone	IV: bolus of 150 mg for stable VT; maintenance: 1 mg/min × 6 h, then 0.5 mg/min × 18 h; PO: 400 mg × q 8–12 h for 7–14 days, then 200–400 mg daily.
Lidocaine	IV: bolus of 1–1.5 mg/kg, can repeat up to total of 3 mg/kg, maintenance: 1–4 mg/min.
Propranolol	IV: 1–3 mg q5 min to a maximum of 5 mg; PO: 10–40 mg q6 h, immediate release; 60–160 mg q12 h, extended release.
Mexiletin	150–300 mg; PO: q8–12 h.
Procainamide	IV: bolus of 10 mg/kg over 20 min, maintenance: 2–3 g/24 h; oral: 500–1250 mg q6 h.
Quinidine	Quinidine sulfate: 200–600 mg; PO: q6–12 h; quinidine gluconate at 324–648 mg; PO: q8 h; IV loading dose: 800 mg/50 mL, maintenance: 50 mg/min.
Sotalol	IV: 7 5 mg q12 h; PO: 80–160 mg q12 h.
Metoprolol	IV: 5 mg q5 min up to 3 doses; PO: metoprolol tartarate at 25–100 mg q12 h.
Esmolol	IV: bolus of 0.5 mg/kg, maintenance: 0.05 mg/kg/min.

## Data Availability

Data are contained within the article.
